# Reporting antimicrobial susceptibility and detection of carbapenemase production in single and double carbapenemase-producing Gram-negative clinical isolates: a nationwide proficiency study

**DOI:** 10.3389/fmicb.2025.1605590

**Published:** 2025-06-10

**Authors:** Felipe Fernández-Cuenca, Mercedes Delgado-Valverde, Pablo Guridi-Fernández, Concepción Gimeno-Cardona, Carmen Hidalgo-Díaz, Álvaro Pascual

**Affiliations:** ^1^Unidad de Gestión Clínica de Enfermedades Infecciosas y Microbiología, Hospital Universitario Virgen Macarena, Sevilla, Spain; ^2^Instituto de Biomedicina de Sevilla (IBIs), Hospital Universitario Virgen Macarena/CSIC/Universidad de Sevilla, Sevilla, Spain; ^3^CIBER de Enfermedades Infecciosas (CIBERINFEC), Instituto de Salud Carlos III, Madrid, Spain; ^4^Departamento de Microbiología, Facultad de Medicina, Universidad de Sevilla, Sevilla, Spain; ^5^Servicio de Microbiología, Consorcio Hospital General Universitario de Valencia, Programa de Control de Calidad CCS-SEIMC, Madrid, Spain

**Keywords:** antimicrobial susceptibility, carbapenemase-producing Gram-negative MDR, national multicenter study, quality control, carbapenemase detection methods

## Abstract

**Purpose:**

The objective was to assess the ability of Spanish clinical microbiology laboratories to report reliable carbapenem susceptibility test results and to detect carbapenemase production (CP) and/or carbapenemase genes in double (DCP) and single carbapenemase-producing (SCP) isolates.

**Methods:**

Twelve isolates (8 SCP and 4 DCP) selected from the Andalusian Reference Laboratory were sent to 83 laboratories with requests for MICs and phenotypic and genotypic tests used for CP.

**Results:**

Overall, there was lower essential agreement and a higher number of clinical errors in DCP than in SCP isolates. Phenotypic tests showed higher sensitivity for DCP isolates than for SCP isolates: lateral flow immunoassay (99.0% vs. 95.1%), carbapenem inactivation method (100% vs. 93%) and chromogenic media (100% vs. 83.3%); conversely, sensitivities for DCP versus SCP isolates was lower using disk diffusion with inhibitors (83.3% vs. 90.4%) and hydrolysis-based assays (81.3% vs. 86.1%). Molecular methods showed higher sensitivity for DCP isolates than phenotypic methods, and higher sensitivity for DCP isolates than for SCP isolates. In addition, concordance between genes detected and the reference was higher in DCP than in SCP isolates (98.9% vs. 83%). However, *bla*VIM-1 and *bla*IMP-8 were not detected in 77.5% and 42.2% of the determinations, respectively, for DCP isolates.

**Conclusion:**

The main differences between DCP versus SCP isolates were the lower reliability of gradient strips, higher overall sensitivity of phenotypic methods for DCP isolates, but lower sensitivity with disk diffusion inhibitors and hydrolysis-based assays. Molecular assays were generally more sensitive for carbapenemase gene detection in DCP than in SCP isolates, although concordance was lower.

## Introduction

Carbapenem resistance in Gram-negative bacilli (GNB) is rapidly increasing and spreading worldwide, with significant clinical, therapeutic and epidemiological repercussions (Paniagua-García et al., 2024). According to data reported by EARS-Net for the EU, the incidence of carbapenem-resistant *Klebsiella pneumoniae* increased by almost 50% between 2019 and 2022, while the incidence of *Pseudomonas aeurginosa* (18%–19%) and *Acinetobacter baumannii* (36%–39%) remained constant during this period (ECDC, 2023). From an epidemiological point of view, the acquisition of plasmid-encoded carbapenemases is the main mechanism of carbapenem resistance (Khaled and Aboshanab, 2020). The most frequently reported belong to molecular classes A (KPC-like), B (VIM-like, IMP-type and NDM-like) and D (OXA-48-like in Enterobacterales). In recent years, there has been a disturbing increase worldwide in the detection of isolates co-producing multiple acquired *β*-lactamases, such as different carbapenemases, extended-spectrum beta-lactamases (ESBL) and/or plasmid-encoded AmpC-type enzymes. The co-production of multiple carbapenemases is rapidly increasing and has been reported worldwide (Tadese et al., 2022). Genes encoding carbapenemases are often linked to other genetic determinants that cause resistance to other antimicrobials (such as colistin, aminoglycosides and/or fosfomycin, among others) and confer multidrug resistance, extensive drug resistance or pandrug resistance. Therefore, infections caused by isolates co-producing several carbapenemases represent a serious clinical and microbiological problem, as well as a serious global problem for human health due to the limited or no therapeutic alternatives available and the high impact of these infections on morbidity, mortality, and healthcare costs (Bonomo et al., 2017).

The detection of carbapenemase production presents important diagnostic, clinical, and epidemiological challenges. In the laboratory, accurate identification can be difficult due to the type of carbapenemase—such as certain OXA-48-type variants with low hydrolytic activity or low expression levels—and the presence of additional resistance mechanisms like porin loss in AmpC-or ESBL-hyperproducing isolates. These factors can lead to missed or delayed detection, resulting in inappropriate treatment, poorer patient outcomes, and increased mortality. From an epidemiological perspective, underdetection compromises surveillance efforts and delays outbreak containment. High-quality testing is therefore essential—not only to guide effective antimicrobial therapy but also to support infection control and limit the spread of resistance within clinical settings.

The EUCAST algorithm for the detection of carbapenemase production includes susceptibility testing of carbapenems, especially ertapenem (high sensitivity) and meropenem (high specificity), as phenotypic markers for carbapenemase production (The European Committee on Antimicrobial Susceptibility Testing, 2017). These results need to be confirmed by phenotypic and molecular assays due to the limitations of ertapenem in terms of specificity. A wide variety of phenotypic methods are currently available for the detection of the most frequent carbapenemases (Tamma and Simner, 2018).

The main objective of this study was to evaluate the ability of Spanish clinical microbiology laboratories to report reliable carbapenem susceptibility test results and to detect carbapenemase production and/or carbapenemase genes in double carbapenemase-producing (DCP) and single carbapenemase-producing (SCP) isolates.

## Methods

### Antimicrobial susceptibility testing and molecular characterization of isolates

For the present study, 12 carbapenemase-producing GNB isolates were selected (see Supplementary Table 1): eight isolates were classified as SCP and 4 as DCP. Eight of the isolates also co-produced one or two ESBLs (CTX-M-15 in 5 isolates and SHV-12 in 3 isolates). The isolates were selected from the reference laboratory of the Andalusian program for the surveillance and control of healthcare-associated infections and antibiotic stewardship (PIRASOA), based at the Hospital Universitario Virgen Macarena, Seville, Spain. Bacterial identification, antimicrobial susceptibility testing and confirmation of carbapenemase production were verified by the PIRASOA clinical microbiology reference laboratory. Isolates were identified by matrix-assisted laser desorption/ionization time-of-flight mass spectrometry (MALDI-TOF MS. Bruker Daltonics, Madrid, Spain). The minimum inhibitory concentration (MIC) of ertapenem, imipenem and meropenem (Supplementary Table 2) were determined in triplicate at the reference center by broth microdilution, according to EUCAST and CLSI guidelines (ISO, 2006; Weinstein, 2022). *Escherichia coli* ATCC 25922 was used as the control strain (The European Committee on Antimicrobial Susceptibility Testing, 2021). The clinical categories were interpreted using EUCAST 2022 breakpoints (EUCAST, 2022).

The *β* Carba™ test (Bio-Rad), a double-disk assay with inhibitors (ROSCO Diagnostics) and lateral flow immunochromatography [NG CARBA-5 (Biotech®)] were used for carbapenemase detection.

Whole Genome Sequencing (WGS) was performed using the Miseq sequencing system (Illumina, San Diego, CA, United States) for the identification of genes encoding carbapenemases, resistome analysis and multilocus sequencing typing (MLST). Genomes were analyzed using the database at https://www.genomicepidemiology.org (ResFinder 4.0 and MLSTFinder 2.0). The main characteristics of the isolates (sequence type, chromosomal and acquired resistome) are shown in Supplementary Table 1.

The isolates were then sent in eSwab® transport medium to 83 participating hospitals in November 2021. Ten centers decided not to participate in the study. 71.2% (52/73) of participating centers were tertiary level hospitals, 4 were privated (5.5%). The laboratory instructions specified that the isolates should be treated as blood culture isolates.

### Study variables

For each isolate, participating laboratories were asked to complete an electronic form indicating: (i) the antimicrobial susceptibility testing system used; (ii) the antimicrobial susceptibility results obtained (MIC values); (iii) the phenotypic method used to detect carbapenemase production; and (iv) the molecular method used to detect carbapenemase genes.

### Analysis of results

The results of the variables below were then analyzed in terms of SCP and DCP isolates: (i) type of carbapenem susceptibility testing method used, (ii) essential agreement (EA) and categorical agreement (CA) using EUCAST 2022 breakpoints as reference (EUCAST, 2022), and categorical error rates (minor errors, major errors and very major errors) (Clark et al., 2019) by type of carbapenem tested (ertapenem, imipenem and meropenem), type of carbapenem susceptibility testing method used, and type of carbapenemase produced; essential and categorical agreements were used to evaluate the performance of a test method compared to the reference method. EA was determined by comparing the MIC results obtained from the test method to those from the reference method. An MIC result was considered to be in essential agreement if it fell within ±1 two-fold dilution of the MIC determined by the reference method. CA was determined by comparing the clinical interpretation—Susceptible (S), Susceptible, Increased Exposure (I), or Resistant (R)—assigned by the test method to that assigned by the reference method. Agreement was considered present when both methods yielded the same category. (iii) detection of carbapenemase production by phenotypic method (number of positive results/total number of results reported); (iv) concordance in the detection of carbapenemase production using phenotypic and genotypic methods, and (v) concordance in the identification of carbapenemase genes by molecular methods, using whole genome sequencing and ResFinder 4.6.0^1^ as reference. The results of the molecular methods obtained by the participating laboratories were considered concordant with those of the reference method when the same carbapenemase family was detected.

### Statistical analysis

Observed differences in these variables between SCP and DCP isolates were analyzed using the chi-square test (or Fisher’s exact test, as appropriate). Differences were considered statistically significant at *p*-values < 0.05.

## Results

### Discrepancies in MICs and categorical error rates

Seventy-three clinical laboratories returned a total of 2,239 MIC analyzable results. Supplementary Table 3 shows the frequency with which the centers employed diffusion and dilution methods. As shown in Table 1, all three carbapenem MICs showed low essential agreement ranging from 72.7% to 89.9%. The lowest essential agreement was for meropenem (SCP 72.7% vs. DCP 74.0%). For ertapenem, essential agreement was higher in DCP isolates (*p* < 0.05), while for imipenem, essential agreement was higher in SCP isolates (*p* < 0.05). Categorical agreement for all carbapenems tested, and percentages of minor errors, major errors and very major errors are shown in Table 1. These errors were more frequently observed with imipenem and meropem among DCP.

**Table 1:** Distribution of MIC discrepancies for carbapenems expressed as essential agreement, categorical agreement and error rates for single carbapenemase-producing (SCP) vs. double carbapenemase-producing (DCP) isolates.

Carbapenem	No of MICs reported (no. of centers)		Essential agreement (%)		Categorical agreement (%)		Categorical errors (%)^**^					
							VME		ME		mE	
	SCP	DCP	SCP	DCP	SCP	DCP	SCP	DCP	SCP	DCP	SCP	DCP
Ertapenem	356 (71)	264 (70)	82.9	89.8^*^	74.4	97.7^*^	17.9	1.5^*^	-	-	0.8	0.8
Imipenem	562 (73)	280 (72)	85.6	75.0^*^	88.3	61.8^*^	3.7	18.6^*^	1.6	-	6.4	19.6^*^
Meropenem	527 (72)	250 (67)	72.7	74.0	66.0	48.4^*^	1.9	-	15.2	8.8^*^	17.3	42.8^*^

^*^p < 0.05. ^**^VME, very major errors; ME, major errors; mE, minor errors.

Regarding the antimicrobial susceptibility testing system used, overall essential agreements ranged 50.8% (gradient strips) to 92.2% (BD Phoenix™), regardless of the isolate group (SCP or DCP) (Table 2).

**Table 2:** Essential and categorical agreement for carbapenems, and categorical error rates for single carbapenemase-producing (SCP) vs. double carbapenemase-producing (DCP) isolates by different antimicrobial susceptibility testing (AST) systems.

AST system	Essential agreement^a^			Categorical agreement			% of categorical errors^b^					
							mEs		MEs		VMEs	
	Overall	SCP	DCP	Overall	SCP	DCP	SCP	DCP	SCP	DCP	SCP	DCP
Gradient strips	50.8	57.8	33.3	62.6	67.2	56.3	**10.3**	**21.8**	**4.3**	**11.5**	**10.3**	**10.3**
Sensititre™	82.8	79.5	90.0	73.4	77.3	70.0	**9.1**	10.0	0	0	**6.8**	**15.0**
Broth microdilution (BMD)	75.0	66.7	**90.9**	59.3	61.9	54.5	**14.3**	**36.4**	**9.5**	**9.1**	**4.8**	**9.1**
VITEK®2	84.5	86.1	79.1	77.1	78.7	73.9	**13.3**	**15.9**	**4.2**	**3.6**	**4.2**	**3.6**
MicroScan WalkAway	83.3	88.7	85.3	74.4	76.5	70.6	**13.8**	**20.4**	0.5	1.2	**7.2**	**7.3**
BD Phoenix™	**92.2**	**92.3**	**91.9**	74.5	80.0	64.9	**12.3**	**29.7**	1.5	2.7	**3.1**	**2.7**

^a^MICs were considered discrepant when the MIC provided by the participating laboratory was not within a single 2-fold dilution (±1 doubling dilution) of the reference MIC result. Essential and categorical agreement > 90% are highlighted in bold. ^b^mEs, minor errors; MEs, major errors; VME, very major errors. Figures in bold show the highest percentages of overall errors, as well as mEs > 10%, MEs > 3%, and VMEs > 1.5% (Clark et al., 2019). The sum of some percentages may exceed 100% when laboratories employed more than one method.

Overall and for both groups of isolates, categorical agreement was less than 90% (62.6% to 77.1%) for all methods used. The method showing the highest number of discrepant MICs and the lowest categorical agreement was the gradient strip technique. For all antimicrobial susceptibility testing systems, minor errors were >10% in both groups, except for Sensititre™ (SCP = 9.1% and DCP = 10%). The number of minor errors using the Sensititre™, Microscan and Phoenix™ systems was acceptable, for both sets of isolates. The percentages of very major errors for SCP and DCP isolates were very high (>1.5%) in all cases (Table 2).

Concordance by type of carbapenemase reported is shown in Table 3. No significant differences were observed between SCP and DCP Enterobacterales. Only *P. aeruginosa* isolates showed an essential agreement >90%. The only isolate exhibiting a 100% of essential agreement was IMP-23-producing *P. aeruginosa* (CC-10). Among SCP isolates, NDM-1-producing *K. pneumoniae* and OXA-48-producing *E. coli* showed the lowest essential agreement. In DCP isolates, those co-producing OXA-48 plus VIM-1 showed the lowest essential agreement (<80%). OXA-48-producing *E. coli* (CC-07) showed the highest rates of discrepant MICs for all carbapenems (ertapenem 23.9%, imipenem 24.6%, and meropenem 31.7%). All isolates except CC-07 and CC-08 showed essential agreement >90% for ertapenem, and all were suboptimal for meropenem in terms of essential agreement. Categorical agreement exceeded 90% in just two isolates (CC-10 and CC-12), both of which were SPC isolates. In general, no differences were detected between SPC and DCP isolates. Minor errors were more frequently detected in DCP isolates, which was mainly due to the susceptibility of CC-04 and CC-08 to increased exposure to meropenem. The CC-11 isolate showed a major error rate of 47.0%; this isolate was susceptible to meropenem, but all centers reported it as resistant. Overall, the number of very major errors detected was high, especially for Enterobacterales isolates, for which ranged from 1.5 to 28.1% (SCP) and from 0.5 to 17.8% (DCP).

**Table 3:** Essential agreement, categorical agreement and errors in single carbapenemase-producing (SCP) and double carbapenemase-producing (DCP) isolates by type of carbapenemase.

Carbapenemase-producing isolates	Type of carbapenemase genes	% Essential agreement^a^	% Categorical agreement^b^			
			Overall	mEs	MEs	VMEs
SCP isolates						
*K. pneumoniae* CC-01	*bla*_IMP-8_	83.1	76.9	**16.4**	-	**6.7**
*K. pneumoniae* CC-02	*bla*_NDM-1_	69.5	68.0	**30.0**	-	1.5
*K. pneumoniae* CC-05	*bla*_KPC-3_	83.7	83.7	5.9	-	**10.4**
*E. coli* CC-06	*bla*_OXA-48_	85.2	84.9	6.3	**5.8**	**1.9**
*E. coli* CC-07	*bla*_OXA-48_	73.5	66.8	3.6	1.5	**28.1**
*P. aeruginosa* CC-10	*bla*_IMP-23_	**100**	**100**	0	-	0
*P. aeruginosa* CC-11	*bla*_VIM-2_	**93.2**	43.3	0	**47.9**	0
*A. baumannii* CC-12	*bla*_NDM-1_	**91.9**	**90.5**	3.4	-	**0**
DCP isolates						
*K. pneumoniae* CC-03	*bla*_VIM-1_, *bla*_OXA-48_	73.3	65.3	**13.9**	2.9	**17.8**
*K. pneumoniae* CC-04	*bla*_VIM-1_, *bla*_KPC-2_	86.4	70.9	**28.6**	-	0.5
*E. cloacae* CC-08	*bla*_VIM-1_, *bla*_IMP-8_	85.7	84.2	**13.3**	-	**2.6**
*C. freundii* CC-09	*bla*_VIM-1_, *bla*_OXA-48_	75.2	58.9	**26.9**	**8.1**	**7.1**

^a^MICs > 1 dilution from the reference values were considered to be discrepant. Essential agreement and categorical agreement > 90% are highlighted in bold. ^b^The highest percentages of overall errors, as well as mEs > 10%, MEs > 3%, and VMEs > 1.5% are highlighted in bold (Clark et al., 2019). The sum of some percentages may exceed 100% when laboratories employed more than one method.

### Phenotypic detection of carbapenemase production

A total of 67 centers (90.5%) provided analyzable results (*n* = 970) for the phenotypic detection of carbapenemase production. The most commonly used phenotypic methods were lateral flow immunochromatography (62.7%; [59% NG CARBA-5, Biotech®; 41.0% Coris BioConcept®]), double-disk inhibition assays (17.4%), the carbapenem inactivation method (6.1%), colorimetric detection of carbapenem hydrolysis (colorimetric assays: 4.1%; [52.5% *β* Carba™, Bio-Rad; 40.0% NEO-Rapid CARB, Rosco Diagnostica; 7.5% in-house methods]) and chromogenic media (4.8%). Other less frequently used methods were the Hodge test (1.4%), temocillin DD (0.8%), MALDI-TOF (0.6%), and ‘other’ (1.3%).

The overall percentage of positive results was 93.5%. Among the most commonly used methods, lateral flow immunochromatography, the carbapenem inactivation method and hydrolysis-based assays had the highest sensitivity (>95%). Among the lateral flow immunochromatography methods, NG CARBA-5 showed a slightly higher, but not significant, sensitivity compared to the Coris BioConcept® assay (98.3% vs. 91.6%). The β Carba™ test was more sensitive than the NEO-Rapid CARB test (100% vs. 87.5%; *p* = 0.05). The least sensitive methods were chromogenic media and double-disk inhibition assays (89.4% and 88.0%, respectively). MALDI-TOF and temocillin DD testing were used by 100% of participants, and 84.6% used the Hodge test.

The phenotypic assays that showed the highest positive results were lateral flow immunochromatography, chromogenic media and the carbapenem inactivation assay regardless of whether were SCP or DCP (Table 4). In contrast, the double-disk inhibition assay and hydrolysis-based assays were less sensitive among DCP than SCP isolates.

**Table 4:** Detection of carbapenemase production in single carbapenemase-producing (SCP) and double-carbapenemase-producing (DCP) isolates using phenotypic tests.

Phenotypic test	Number of positive results/Total number tested (%)^a^														
	SCP isolates									DCP isolates					*p*
	CC-01	CC-02	CC-05	CC-06	CC-07	CC-10	CC-11	CC-12	Total (%)	CC-03	CC-04	CC-08	CC-9	Total (%)	
	*bla*_IMP-8_	*bla*_NDM-1_	*bla*_KPC-3_	*bla*_OXA-48_	*bla*_OXA-48_	*bla*_IMP-23_	*bla*_VIM-2_	*bla*_NDM-1_		*bla*_VIM-1_ *bla*_OXA-48_	*bla*_VIM-1_ *bla*_KPC-2_	*bla*_VIM-1_ *bla*_IMP-8_	*bla*_VIM-1_ *bla*_OXA-48_		
Lateral flow immunochromatography	49/53 (92.5)	51/53 (96.2)	47/49 (95.9)	49/49 (100)	42/44 (95.5)	48/52 (92.3)	47/47 (100)	**37/42 (88.1)**	389/370 (95.1)	53/53 (100)	49/49 (100)	50/52 (96.1)	53/53 (100)	205/207 (99.0)	**<0.05**
Disk diffusion with inhibitors	**18/21 (85.7)**	17/18 (94.4)	11/11 (100)	**11/15 (73.3)**	**6/8 (75.0)**	15/16 (93.8)	16/16 (100)	10/10 (100)	104/115 (90.4)	**10/15 (66.7)**	**13/16 (81.3)**	15/15 (100)	**12/14 (85.7)**	**50/60 (83.3)**	0.17
Chromogenic medium	**3/4 (75.0)**	4/4 (100)	4/4 (100)	**3/4 (75.0)**	**2/4 (50.0)**	**3/4 (75.0)**	3/3 (100)	3/3 (100)	**25/30 (83.3)**	4/4 (100)	5/5 (100)	4/4 (100)	4/4 (100)	17/17 (100)	0.07
Carbapenem inactivation assay	6/6 (100)	7/7 (100)	**6/7 (85.7)**	**6/7 (85.7)**	**4/5 (80.0)**	4/4 (100)	4/4 (100)	3/3 (100)	40/43 (93.0)	7/7 (100)	4/4 (100)	5/5 (100)	5/5 (100)	21/21 (100)	**<0.05**
Hydrolysis-based method	**2/3 (66.7)**	4/4 (100)	6/6 (100)	**4/5 (80.0)**	**0/1 (0)**	**6/7 (85.7)**	**5/6 (83.3)**	4/4 (100)	**31/36 (86.1)**	**3/4 (75.0)**	5/5 (100)	**2/3 (66.7)**	**3/4 (75.0)**	**13/16 (81.3)**	0.7
Temocillin disk	NA	NA	NA	1/1 (100)	1/1 (100)	NA	NA	NA	2/2 (100)	1/1 (100)	NA	NA	1/1 (100)	2/2 (100)	NA
Hodge test	2/2 (100)	1/1 (100)	NA	1/1 (100)	**0/1 (0)**	2/2 (100)	1/1 (100)	**0/1 (0)**	**7/9 (77.8)**	1/1 (100)	1/1 (100)	1/1 (100)	1/1 (100)	4/4 (100)	NA
MALDI-TOF	1/1 (100)	1/1 (100)	1/1 (100)	NA	NA	NA	1/1 (100)	1/1 (100)	5/5 (100)	NA	1/1 (100)	NA	NA	1/1 (100)	NA

^a^The lowest percentages of detection are in bold. The sum of some percentages may exceed 100% when laboratories employed more than one method.

With respect to the type of carbapenemase co-produced, using phenotypic methods (see Table 4; Supplementary Table 4) the overall rate of positive detection of carbapenemase production was slightly higher among DCP isolates than SCP isolates (95.2% vs. 92.7%, *p* = 0.1). Among SCP isolates, a higher percentage of false negative results was observed with the non-ESBL OXA-48-producing (15.1%) and IMP-8-producing (11.0%) isolates. However, among DCP isolates, the lowest percentage of false negative results was observed for the VIM-1, OXA-48 and CTX-M-15 co-producing isolate (91.9%).

An analysis was performed using two pairs of isolates to determine the effect of ESBL production on phenotypic detection of carbapenemase production in SCP isolates. The first pair were VIM-1-producing isolates: one was a CTX-M-15-and SHV-12-producing *K. pneumoniae* and the other a non-ESBL-producing *A. baumannii* (89.0 and 90.8% of positive results, respectively; *p* = 0.12). The second pair were OXA-48 producers: one a CTX-M-15-producing *K. pneumoniae* and the other a non-ESBL-producing *E. coli* (91.6% and 84.9% of positive results, respectively; *p* = 0.2). Among DCP isolates, the percentages of positive results in the two VIM-1 + OXA-48-co-producing isolates (one also producing CTX-M-15 and the other also producing SHV-12) were 91.9% and 96.4%, respectively (*p* = 0.2).

### Molecular detection of carbapenemase genes

The most frequently used multiplex real-time PCR (mRT-PCR, 49.5%) was the Xpert Carba-R (GeneXpert-Cepheid; 59.8%), followed by the Allplex™ Entero-DR Assay (Seegene; 28.6%), the RealCycler® OXVIKP (Progenie Molecular; 4.5%), Unyvero i60 ITI® (Curetis, 4.5%), and Verigene® (Nanosphere; 2.6%). Two PCR and array hybridization (PCR-hyb; 20.3%) methods were used: the AMR Direct Flow Chip assay (Master Diagnostic; 93.6%) and the FilmArray® Panel (BioFire Diagnostics; 6.4%). Only one loop-mediated isothermal amplification (LAMP) assay was used (Eazyplex® SuperBug CRE system; 15.8%). Other methods used included in-house PCR (13.6%) and WGS using the Illumina NGS platform (0.7%).

The most commonly used molecular methods are shown in Table 5. The overall concordances were statistically significantly lower for DCP than for SCP isolates using the Eazyplex Superbug CRE system (60.7% vs. 80.7%, respectively; *p* < 0.05) and the Xpert Carba-R (57.4% vs. 73.3%, respectively; *p* = 0.04). In contrast, no significant differences were observed when comparing DCP and SCP isolates using the AMR Direct Flow Chip (89.3% vs. 86.4%, respectively; *p* = 0.7), the Allplex Entero-DR Assay (76.9% vs. 74.0%, respectively; *p* = 0.3), and in-house PCRs (75.0% vs. 89.8%, respectively; *p* = 0.1).

**Table 5:** Concordance in the detection of carbapenemase-encoding genes in single carbapenemase-producing (SCP) and double carbapenemase-producing (DCP) isolates using molecular methods.

Molecular method	Number of concordant results/ Total number tested by this method (%)^a^														*p*
	SCP isolates									DCP isolates					
	CC-01	CC-02	CC-05	CC-06	CC-07	CC-10	CC-11	CC-12	Total (%)	CC-03	CC-04	CC-08	CC-9	Total (%)	
	*bla*_IMP-8_	*bla*_NDM-1_	*bla*_KPC-3_	*bla*_OXA-48_	*bla*_OXA-48_	*bla*_IMP-23_	*bla*_VIM-2_	*bla*_NDM-1_		*bla*_VIM-1_ *bla*_OXA-48_	*bla*_VIM-1_ *bla*_KPC-2_	*bla*_VIM-1_ *bla*_IMP-8_	*bla*_VIM-1_ *bla*_OXA-48_		
Eazyplex® SuperBug CRE system	**3/8 (37.5)**	8/8 (100)	7/7 (100)	**6/7 (85.7)**	**5/6 (83.3)**	**4/7 (57.1)**	6/6 (100)	**7/8 (87.5)**	**46/57 (80.7)**	**0/5 (0)**	8/8 (100)	**1/7 (14.3)**	8/8 (100)	**17/28 (60.7)**	**0.05**
FilmArray® (BioFire Diagnostic)	1/1 (100)	0 (0)	2/2 (100)	1/1 (100)	0 (0)	2/2 (100)	0 (0)	0 (0)	6/6 (100)	0 (0)	1/1 (100)	0 (0)	0 (0)	1/1 (100)	NA
In-house PCR	**4/6 (66.7)**	6/6 (100)	6/6 (100)	6/6 (100)	6/6 (100)	**3/6 (50.0)**	6/6 (100)	**6/7 (85.7)**	**44/49 (89.8)**	**2/6 (33.3)**	6/6 (100)	**4/6 (66.7)**	6/6 (100)	**18/24 (75.0)**	0.1
WGS (Miseq)	1/1 (100)	1/1 (100)	0 (0)	0 (0)	0 (0)	0 (0)	0 (0)	0 (0)	2/2 (100)	0 (0)	1/1 (100)	0 (0)	1/1 (100)	2/2 (100)	NA
RealCycler® OXVIKP (Progenie Molecular)	1/1 (100)	1/1 (100)	1/1 (100)	1/1 (100)	1/1 (100)	0/1 (0)	1/1 (100)	1/1 (100)	**7/8 (87.5)**	0/1 (0)	0/1 (0)	0/1 (0)	1/1 (100)	**1/4 (25.0)**	NA
Allplex™ Entero-DR Assay (Seegene)	**5/6 (83.3)**	6/6 (100)	**6/7 (85.7)**	**4/6 (66.7)**	**5/6 (83.3)**	**5/7 (71.4)**	6/6 (100)	6/6 (100)	**43/50 (74.0)**	**0/5 (0)**	**6/7 (85.7)**	7/7 (100)	7/7 (100)	**20/26 (76.9)**	0.3
Verigene® (Nanosphere)	0 (0)	0 (0)	1/1 (100)	0 (0)	0 (0)	1/1 (100)	1/1 (100)	1/1 (100)	4/4 (100)	0/1 (0)	0 (0)	0/1 (0)	1/1 (100)	**1/3 (33.3)**	NA
AMR Direct Flow Chip (Master Diagnostic)	**8/10 (80.0)**	8/8 (100)	**4/8 (50.0)**	8/8 (100)	7/7 (100)	8/8 (100)	**7/8 (87.5)**	**7/9 (77.8)**	**57/66 (86.4)**	**0/8 (0)**	8/8 (100)	**9/10 (90.0)**	**8/10 (80.0)**	**25/28 (89.3)**	0.7
Xpert Carba-R (Genexpert-Cepheid)	**1/13 (7.7)**	15/15 (100)	11/11 (100)	12/13 (92.3)	9/9 (100)	**0/13 (0)**	15/16 (93.8)	14/15 (93.3)	**77/105 (73.3)**	**2/13 (15.4)**	**14/15 (93.3)**	**2/12 (16.7)**	**13/14 (92.9)**	**31/54 (57.4)**	**0.04**
Unyvero i 60 ITI	1/1 (100)	1/1 (100)	0/1 (0)	1/1 (100)	1/1 (100)	1/1 (100)	1/1 (100)	1/1 (100)	**7/8 (87.5)**	**0/1 (0)**	1/1 (100)	1/1 (100)	1/1 (100)	**3/4 (75.0)**	NA

Percentages of positive results < 90% are in bold. NA, not applicable. *p*, statistically significant differences between SCP and DCP detection. The sum of some percentages may exceed 100% when laboratories employed more than one method.

With respect to the type of carbapenemase genes co-produced (Supplementary Table 4), the overall rates of positive concordance were higher for DCP isolates than for SCP isolates (98.9% vs. 83.0%, respectively; *p* < 0.05), whereas overall concordance in the detection of carbapenemase genes was lower for DCP isolates than for SCP isolates (65.2% vs. 87.9%, respectively; *p* < 0.05). Further analysis showed that for *K. pneumoniae* isolates carrying both *bla*_VIM-1_ and *bla*_OXA-48_, the PCR results were positive only for *bla*_OXA-48_, and that *bla*_VIM_ was not detected at all in 77.5% of the results analyzed. With respect to isolates co-harboring *bla*_IMP-8_ plus *bla*_VIM-1_, the *bla*_IMP-8_ gene was not detected in 42.2% of the results analyzed.

## Discussion

The multiple carbapenemase-producing isolates that are emerging worldwide have potentially worrying clinical, epidemiological and microbiological implications (Meletis et al., 2015). The present study is, to the best of our knowledge, the first to focus on DCP and SCP isolates to determine whether interpretation of antimicrobial susceptibility results (discrepant MICs and errors) and detection of carbapenemase genes are more difficult in DCP isolates than in SCP isolates.

We noted some relevant findings in our study that may be difficult to compare with the results of previous studies that included carbapenemase-producing GNB isolates because differences between DCP and SCP isolates were not investigated.

For antimicrobial susceptibility test results, the gradient strip method was found to be the least reliable. This method returned unacceptably high percentages of discrepant MICs and very major errors (false susceptible), which is consistent with previously published findings (Lee and Chung, 2015; Markelz et al., 2011). There were significant differences between DCP and SCP isolates, suggesting that the interpretation of antimicrobial test results is less reliable for DCP compared to SCP isolates, with an unacceptably high percentage of discrepant MICs, lower categorical agreement, and errors, particularly very major errors with imipenem, and minor errors with imipenem and meropenem. However, a high number of very major errors were detected for ertapenem in SCP isolates. The reasons for this were not investigated, but may be explained by methodological factors related to inoculum preparation, decreased antimicrobial activity, or interpretation of MIC values, especially when small bacterial subpopulations are growing within the inhibition halo (heteroresistance). Heteroresistant subpopulations can be difficult to detect (Humphries et al., 2018; Fernández-Cuenca et al., 2021; Harino et al., 2013), as has been observed in porin-deficient *K. pneumoniae* isolates harboring *bla*_OXA-48_, or isolates carrying *bla*_VIM-type_ or *bla*_KPC-type_ genes (Tato et al., 2010; Nodari et al., 2015; Adams-Sapper et al., 2015; López-Camacho et al., 2019).

When false resistance (major error) to meropenem happens, the problem can be resolved by testing with a second, more reliable antimicrobial susceptibility testing method. False susceptibility to ertapenem and imipenem (very major errors) on the other hand often goes undetected because susceptible results are not routinely checked in the laboratory. It is essential therefore that laboratories have the ability to detect both types of false results when testing carbapenems in order to avoid potentially serious consequences for the management and outcome of patients infected with DCP isolates, as has been described for SCP isolates. These consequences include treatment failure associated with increased mortality and prolonged length of stay (Bartoletti et al., 2022) following false susceptible results, or missing a possible good opportunity to use carbapenems, in accordance with EUCAST recommendations, following false resistance results. Carbapenemase production can usually be inferred from carbapenem susceptibility results, so that it is essential that antimicrobial susceptibility testing is reliable (The European Committee on Antimicrobial Susceptibility Testing, 2017; Markelz et al., 2011; Kanahashi et al., 2021).

The second major objective of our study was to assess the ability to detect carbapenemase production in SCP and DCP isolates. In principle, a higher rate of detection of positive results would be expected among DCP isolates than SCP isolates on the grounds that, regardless of gene expression or gene copy number, the amount of carbapenemase produced in DCP isolates is likely to be higher due to the presence of two carbapenemase-encoding genes. In our study, this hypothesis was true for lateral flow immunochromatography and the carbapenem inactivation assay, but not for the double-disk assay, hydrolysis-based assays and chromogenic media. This is a very important finding and suggests that lateral flow immunochromatography or carbapenem inactivation assays should be included in the algorithms currently used for detection of carbapenemases. Double-disk inhibition assays and hydrolysis-based assays returned unexpectedly high rates of false negative results in both DCP and SCP isolates. There are several possible explanations for this. First, use of the double-disk inhibition assay without the temocillin disk is highly sensitive, but has low specificity for phenotypic detection of *bla*_OXA-48._ Second, methodological problems (such as inoculum preparation at low bacterial concentrations) or the interpretation of results (e.g., color changes using hydrolysis-based assays with colorimetric detection) (Kanahashi et al., 2021). Third, the type of carbapenemase produced, especially *bla*_OXA-48_, due to the low catalytic efficiency of this group of carbapenemases, as reported previously (Österblad et al., 2014), and IMP-type carbapenemases, especially *bla*_IMP-8_, which is consistent with other studies showing the potential difficulty of detecting this heterogeneous group of class B carbapenemases (Jenkins et al., 2020; Findlay et al., 2015).

In our study, the presence of an ESBL or type of ESBL (*bla*_CTX-M-15_ or *bla*_SHV-12_) did not affect the detection of carbapenemase production, regardless of whether or not a carbapenemase was co-produced. Nevertheless, this finding should be interpreted with caution due to the small number of ESBL-producing isolates included. Furthermore, the possibility cannot be ruled out. The presence of ESBLs in isolates with significant efflux pump overexpression and lacking major porins involved in carbapenem resistance may hinder the detection of carbapenemase production, especially with certain carbapenemase gene variants with weak hydrolyzing activity (Hamzaoui et al., 2018). Further experiments with laboratory-derived mutants would help to elucidate the true role of ESBL production in carbapenemase detection.

In our study, genotypic tests showed a higher overall sensitivity for DCP isolates than phenotypic assays, which was consistent with many previous studies (Findlay et al., 2015; Baeza et al., 2019). However, the overall concordance for carbapenemase gene detection was lower in DCP isolates than in SCP isolates, mainly due to the poor detection of *bla*_VIM-1_ and *bla*_IMP-8_ genes. In the case of isolates co-producing *bla*_VIM-1_ and *bla*_OXA-48_, we did not exclude the possibility of loss of the plasmid harboring *bla*_VIM-1_, as most assays failed to detect this gene. Loss of plasmid genes encoding carbapenemases or other types of *β*-lactamases has been reported previously and appears to be rare (Hopkins et al., 2018). A recent study found that the plasmid loss rate in *bla*_OXA-like_-producing *Klebsiella* spp. and *Enterobacter cloacae* was 4.3% and 12.4% (Mahazu et al., 2022).

Of the assays that were most frequently performed, the least reliable were the LAMP Eazyplex® SuperBug CRE assay, followed by the mRT-PCR Xpert Carba-R assay, which showed lower concordance with the reference in DCP versus SCP isolates. This was an unexpected result and may be related to certain carbapenemase genes being more difficult to detect with these assays than others, as has been reported for some IMP-type carbapenemase variants, including *bla*_IMP-8_ (Kanahashi et al., 2021; Huang et al., 2022; Lowe et al., 2020). Yu-Tsung et al. tested a collection of carbapenemase-producing isolates, and the only negative result they obtained with both NG-CARBA 5 and Xpert Carba-R was with a *K. pneumoniae* isolate co-producing VIM-1 and IMP-8 (Huang et al., 2022).

This study has several minor limitations. First, the small number of isolates, although this is not usual for this type of study. Second, the source of discrepant MICs and errors was not investigated, as this was not an objective of the study. Third, the role of other mechanisms of resistance to carbapenems (such as AmpC-hyperproduction and/or absence of porins and/or overexpression of efflux pumps), which could better explain some of the discrepancies observed, but these resistance mechanisms were not characterized in the isolates selected for this study. Fourth, we did not request information on the working protocols or algorithms used at each center for the detection of carbapenemase production and it cannot be ruled out that some centers used a different method for detection of carbapenemase production with the reference isolates than with isolates routinely collected in their laboratories.

In conclusion, our study demonstrates that discrepancies in MICs and errors are related to the AST system used, especially the gradient strip, and to the carbapenem used, and result in unacceptable very major errors and major errors. For the detection of carbapenemase production, phenotypic assays are more sensitive for DCP isolates than for SCP isolates; the most sensitive assays were lateral flow immunochromatography and the carbapenem inactivation assay, and the genes most challenging for phenotypic detection of carbapenemase production were *bla*_OXA-48_ and the two IMP-type carbapenemases, *bla*_IMP-8_ and *bla*_IMP-23_, especially using double-disk inhibition assays and hydrolysis-based assays. Detection of certain carbapenemase genes, especially *bla*_VIM-1_ and *bla*_IMP-8_, in DCP isolates is unreliable using the LAMP assay, the Eazyplex® SuperBug CRE, and the mRT-PCR Xpert Carba-R.

## Data Availability

The original contributions presented in the study are publicly available. This data can be found at the links below: https://www.ebi.ac.uk/ena/browser/view/PRJEB53686, https://www.ncbi.nlm.nih.gov/bioproject/?term=PRJNA1104021, https://www.ncbi.nlm.nih.gov/bioproject/?term=PRJNA1040118, https://www.ncbi.nlm.nih.gov/bioproject/?term=PRJNA1262334.

## Footnotes

^1^ https://www.genomicepidemiology.org/
