# Supplementary material for: Reporting antimicrobial susceptibility and detection of carbapenemase production in single and double carbapenemase-producing Gram-negative clinical isolates: a nationwide proficiency study
Source: Front Microbiol. 2025 Jun 10;16:1605590. doi: 10.3389/fmicb.2025.1605590 (PMC12185507; doi:10.3389/fmicb.2025.1605590)
Supplement: Supplementary file 1 [file Table_1.docx]

**Supplementary Table 1.** Sequence type (ST) and resistome (acquired and chromosomal) of the 12 carbapenemase-producing Gram-negative bacterial isolates used as reference.

| **Isolate**  **(ST)** | ***bla* genes coding**  **for** | |  | **Acquired resistome** | | | | | |  | **Chromosomal resistome** | | |
| --- | --- | --- | --- | --- | --- | --- | --- | --- | --- | --- | --- | --- | --- |
|  | **CP** | **ESBL** |  | **AG** | **FQ** | **FOS** | **TET** | **TRI** | **SUL** |  | **Efflux pumps** | **AND gyrase** | **Porins** |
| *K. pneumoniae* CC-01 (ST392) | *IMP-8* | *CTX-M-15* |  | *aacA4, aac(6')Ib-cr, aadA1*  *aph(3'')-Ib, aph(6)-Id* | *oqxA, oqxB, aac(6')-Ib-cr, qnrB1* | *fosA* | *Tet(A)* | *dfrA14* | *sul2* |  | *acrR* | *gyrA*  *parC* | *OmpK36*  *OmpK37* |
| *K. pneumoniae* CC-02 (ST147) | *NDM-1* | *CTX-M-15*  *SHV-12* |  | *aac(6')-Ib-cr, aac(3)-IId, aph(3'')-Ib, aph(3')-VI, aph(6)-Id* | *oqxA, oqxB, aac(6')-Ib-cr, qnrB32, qnrB9* | *fosA* | *Tet(A)* | *dfrA1*  *dfrA14* | *sul1* |  | *acrR* | *gyrA*  *parC* | *OmpK36*  *OmpK37* |
| *K. pneumoniae* CC-03 (ST15) | *VIM-1*  *OXA-48* | *CTX-M-15* |  | *aadA2, aac(6')-Ib-cr, aph(3'')-Ib, aph(6)-Id* | *oqxA, oqxB, aac(6')-Ib-cr* | *fosA* | ND | *dfrA12* | *sul1sul2* |  | *acrR* | *gyrA*  *parC* | *OmpK36*  *OmpK37* |
| *K. pneumoniae*  CC-04 (ST11) | *KPC-2*  *VIM-1* | *ND* |  | *aadA1, aadA2, ant(3'')-Ia, aph(3')-XV* | *oqxA, oqxB, aac(6')-Ib-cr,*  *qnrB2* | *fosA* | ND | ND | *sul1* |  | *acrR* | *gyrA*  *parC* | *OmpK36*  *OmpK37* |
| *K. pneumoniae* CC-05 (ST307) | *KPC-3* | *CTX-M-15* |  | *aph(6)-Id, aph(3'')-Ib* | *oqxB, oqxA, aac(6')-Ib-cr,*  *qnrB1* | *fosA* | *Tet(A)* | *dfrA14* | *sul2* |  | *acrR* | *gyrA*  *parC* | *OmpK36*  *OmpK37* |
| *E. coli*  CC-06 (ST405) | *OXA-48* | *CTX-M-15* |  | *aac(3)-IId, aadA5* | *aac(6')-Ib-cr* | ND | *tet(B)* | *dfrA17* | *sul1* |  | ND | *gyrA*  *parC, parE* | ND |
| *E. coli*  CC-07 (ST58) | *OXA-48* | *ND* |  |  |  |  |  |  |  |  |  |  |  |
| *E. cloacae*  CC-08 (ST242) | *IMP-8*  *VIM-1* | *SHV-12* |  | *aadA1, aac(6')-Ib3, aph(3'')-Ib, aph(6)-Id, aph(3')-XV* | *aac(6')-Ib-cr*  *qnrA1* | *fosA* | ND | ND | *sul1 sul2* |  | NA | NA | NA |
| *C. freundii*  CC-09 (ST221) | *VIM-1*  *OXA-48* | *SHV-12* |  | *aadA1, aac(6')-Ib3, aac(3)-IId, aph(3')-XV* | *aac(6')-Ib-cr*  *qnrS1* | ND | ND | ND | *sul1* |  | NA | NA | NA |
| *P. aeruginosa*  CC-10 (ST175) | *IMP-23* | ND |  | *aac(6')-Ib-cr, aac(6')-Ib3, ant(2'')-Ia, aph(3')-II* | *aac(6')-Ib-cr* | ND | ND | ND | *sul1* |  | NA | NA | NA |
| *P. aeruginosa*  CC-11 (ST175) | *VIM-2* | ND |  | *aadA13, aac(6')-Il, ant(2'')-Ia, aph(3')-IIb* | ND | fosA | ND | ND | *sul1* |  | NA | NA | NA |
| 1. *baumannii*   CC-12 (ST85) | *NDM-1* | ND |  | *ant(2'')-Ia* | ND | ND | ND | ND | *sul2* |  | NA | NA | NA |

CP: carbapenemase; ESBL: extended-spectrum β-lactamase.

AG: aminoglycosides; FQ: fluoroquinolones; FOS: fosfomycin; TET: tetracyclines; TRI: trimethoprim; SUL: sulfonamide.

ND: not detected.

NA: not available.

**Supplementary Table 2.** MICs (mg/L) of carbapenems against single carbapenemase-producing and double carbapenemase-producing isolates.

| **Antimicrobial^a^** | **Single carbapenemase producers** | | | | | | | |  | **Double carbapenemase producers** | | | |
| --- | --- | --- | --- | --- | --- | --- | --- | --- | --- | --- | --- | --- | --- |
|  | ***bla*_IMP-8_** | ***bla*_NDM-1_** | ***bla*_KPC-3_** | ***bla*_OXA-48_** | ***bla*_OXA-48_** | ***bla*_IMP-23_** | ***bla*_VIM-2_** | ***bla*_NDM-1_** |  | ***bla*_VIM-1_**  ***bla*_OXA-48_** | ***bla*_VIM-1_**  ***bla*_KPC-2_** | ***bla*_VIM-1_**  ***bla*_IMP-8_** | ***bla*_VIM-1_**  ***bla*_OXA-48_** |
|  | **CC-01** | **CC-02** | **CC-05** | **CC-06** | **CC-07** | **CC-10** | **CC-11** | **CC-12** |  | **CC-03** | **CC-04** | **CC-08** | **CC-09** |
| Ertapenem | **>4** | **>4** | **>4** | **>4** | **1** | NA | NA | NA |  | **>4** | **>4** | **2** | **>4** |
| Imipenem | **4** | **8** | **>8** | 2 | 2 | **>16** | **16** | **>16** |  | **8** | **8** | **8** | **4** |
| Meropenem | **16** | 4 | **>16** | 2 | 0.125 | **>16** | 8 | **>16** |  | 2 | 8 | 4 | 2 |

MICs shown in bold correspond to the R (resistant) clinical category using EUCAST breakpoints, version 12.0.

**Supplementary Table 3.** Methods used by the participating laboratories.

| **Methods used by the participating laboratories** | **Number of centers** | **Number of test** |
| --- | --- | --- |
| **Diffusion methods** |  |  |
| Gradient strip | 24 | 437 |
| **Dilution methods** |  |  |
| In house broth microdilution | 10 | 289 |
| Microscan | 50 | 8761 |
| Phoenix | 5 | 564 |
| Sensititre | 10 | 285 |
| Vitek | 23 | 2982 |

**Supplementary Table 4.** Concordance in the detection of carbapenemase production using phenotypic and genotypic methods in single carbapenemase-producing (SCP) and double carbapenemase-producing (DCP) isolates.

| **Carbapenemase-producing**  **isolates** |  | **Genes encoding** | |  | **No. of positive results /**  **No. of results (%)^a^** | | **Concordance^b^** |
| --- | --- | --- | --- | --- | --- | --- | --- |
|  |  | **Carbapenemases** | **ESBLs** |  | **Phenotypic methods** | **Molecular**  **methods** |  |
| **SCP isolates** |  |  |  |  |  |  |  |
| *K. pneumoniae* CC-01 |  | *bla*_IMP-8_ | *bla*_CTX-M-15_ |  | 81/91 (**89.0**) | 36/58 (**62.1**) | 24/36 (**66.7**) |
| *K. pneumoniae* CC-02 |  | *bla*_NDM-1_ | *bla*_CTX-M-15,_ *bla*_SHV-12_ |  | 86/89 (96.6) | 46/46 (100) | 46/46 (100) |
| *K. pneumoniae* CC-05 |  | *bla*_KPC-3_ | *bla*_CTX-M-15_ |  | 75/78 (96.2) | 39/49 (**79.6**) | 37/39 (94.9) |
| *E. coli* CC-06 |  | *bla*_OXA-48_ | *bla*_CTX-M-15_ |  | 76/83 (**91.6**) | 41/45 (**91.1**) | 37/41 (90.2) |
| *E. coli* CC-07 |  | *bla*_OXA-48_ | none |  | 56/66 (**84.9**) | 36/36 (100) | 32/36 (88.9) |
| *P. aeruginosa* CC-10 |  | *bla*_IMP-23_ | none |  | 81/88 (**92.0**) | 32/60 (**53.3**) | 24/32 (**75.0**) |
| *P. aeruginosa* CC-11 |  | *bla*_VIM-2_ | none |  | 79/80 (98.8) | 45/45 (100) | 44/45 (97.8) |
| *A. baumannii* CC-12 |  | *bla*_NDM-1_ | none |  | 59/65 (90.8) | 47/49 (96.0) | 39/47 (**83.0**) |
| **DCP isolates** |  |  |  |  |  |  |  |
| *K. pneumoniae* CC-03 |  | *bla*_VIM-1_, *bla*_OXA-48_ | *bla*_CTX-M-15_ |  | 79/86 (**91.9**) | 40/40 (100) | 4/40 (**10.0**) |
| *K. pneumoniae* CC-04 |  | *bla*_VIM-1,_ *bla*_KPC-2_ | none |  | 78/81 (96.3) | 48/48 (100) | 45/48 (**93.8**) |
| *E. cloacae* CC-08 |  | *bla*_VIM-1,_ *bla*_IMP-8_ | *bla*_SHV-12_ |  | 77/80 (96.3) | 44/46 (**95.7**) | 24/44 (**54.5**) |
| *C. freundii* CC-09 |  | *bla*_VIM-1_, *bla*_OXA-48_ | *bla*_SHV-12_ |  | 80/83 (96.4) | 49/49 (100) | 45/49 (**91.8**) |

^a^The lowest percentages of carbapenemase detection (<95%) are in bold.

^b^Detection of the same type of carbapenemase(s) using molecular methods. Expressed as a percentage of the total number of positive results.
